# HUNK phosphorylates EGFR to regulate breast cancer metastasis

**DOI:** 10.1038/s41388-019-1046-5

**Published:** 2019-10-09

**Authors:** Carly B. Williams, Kendall Phelps-Polirer, Ivan P. Dingle, Christina J. Williams, Matthew J. Rhett, Scott T. Eblen, Kent Armeson, Elizabeth G. Hill, Elizabeth S. Yeh

**Affiliations:** 1 Department of Cell and Molecular Pharmacology and Experimental Therapeutics, Medical University of South Carolina, 173 Ashley Ave, BSB 358, MSC509, Charleston, SC 29425 USA; 2 Department of Public Health Sciences, Medical University of South Carolina, Charleston, SC 29425 USA; 3 Department of Pharmacology and Toxicology, Indiana University School of Medicine, Simon Cancer Center, Indianapolis, IN 46220 India

**Keywords:** Mechanisms of disease, Breast cancer

## Abstract

Epidermal growth factor receptor (EGFR) is commonly over-expressed in metastatic breast cancer yet metastatic breast cancer is generally resistant to anti-EGFR therapies, and the mechanism for resistance to EGFR inhibitors in this setting is not fully understood. Hormonally up-regulated neu-associated kinase (HUNK) kinase is up-regulated in aggressive breast cancers and is thought to play a role in breast cancer metastasis. However, no studies have been conducted to examine a relationship between EGFR and HUNK in breast cancer metastasis. We performed a kinase substrate screen and identified that EGFR is phosphorylated by HUNK. Our studies show that HUNK phosphorylates EGFR at T654, enhancing receptor stability and downstream signaling. We found that increased phosphorylation of T654 EGFR correlates with increased epithelial to mesenchymal, migration and invasion, and metastasis. In addition, we found that HUNK expression correlates with overall survival and distant metastasis free survival. This study shows that HUNK directly phosphorylates EGFR at T654 to promote metastasis and is the first study to show that the phosphorylation of this site in EGFR regulates metastasis.

## Introduction

The over-expression of epidermal growth factor receptor (EGFR) is associated with poor clinical breast cancer outcomes including early recurrence, increased risk of metastasis, and decreased survival [1–5]. However, the efficacy of anti-EGFR therapies (e.g., cetuximab and erlotinib) in clinical trials was limited [2, 6, 7]. Cetuximab was tested in a phase II trial of 31 patients with triple negative breast cancer (TNBC), and only 2/31 patients responded to treatment, despite the majority of tumors exhibiting over-expression of EGFR [6]. Likewise, erlotinib was tested in a phase II trial in 69 patients with metastatic breast cancer and only 2/69 had a partial response, while 67/69 patients had no response [2]. Consequently, additional studies are needed to understand the underlying mechanistic connection between EGFR expression and metastatic progression.

Experimental studies support the observation that over-expression of EGFR promotes metastatic phenotypes in human breast cancer cells [1, 4, 5, 8–15]. In one study, Appert-Collin et al. found that ligand-independent, constitutively active forms of the receptor can promote metastatic phenotypes of tumor cells [8]. In another study, Xue et al. found that over-expression of EGFR in mammary tumors resulted in increased lung metastases compared with mammary tumors that do not over-express EGFR [5]. Other studies showed that cells with elevated EGFR expression had increased hyperinvasive capabilities compared with cells with reduced EGFR [16]. Importantly, breast cancers that over-express EGFR and exhibit increased metastatic potential, are resistant to EGFR inhibitors [1, 17].

Hormonally Up-regulated Neu-associated Kinase (HUNK) is a serine/threonine kinase that is a member of the AMP-activated protein kinase (AMPK) family, and was reported to be up-regulated in aggressive subsets of human cancers including breast, ovarian, and colon cancers [18]. Experimental studies indicate a relationship between HUNK and breast cancer progression [18–22]. Wertheim et al. showed that *Hunk* wild-type (*Hunk*^*+/+*^) mice had significantly increased lung metastases compared with *Hunk*-knockout (*Hunk*^−*/*−^*)* mice when bred into an MMTV-*myc* background [18]. In addition, tumors derived from *Hunk*^−*/*−^ cells retrovirally transduced with a wild-type version of HUNK (HUNK WT) had significantly increased lung metastases compared with those transduced with either a kinase dead version of HUNK (K91M) or a control vector [18]. These data strongly suggest that HUNK promotes breast cancer metastasis, and that this process is dependent on HUNK kinase activity. However, another study provided evidence that HUNK suppresses basal breast cancer metastasis [23]. Quintela-Fandino et al. found that when HUNK was exogenously introduced into basal breast cancer cells, cell motility and tumor metastases were decreased [23]. With these conflicting views on HUNK’s role in breast cancer metastasis, further investigation is needed.

Currently, a mechanism for HUNK-mediated metastasis has not been delineated. Furthermore, there are no known substrates of HUNK. Identification of bona fide substrates could give more insight into HUNK’s intracellular functions, particularly in breast cancer. In this study, we show that HUNK directly phosphorylates EGFR in its juxtamembrane domain at threonine 654 (T654), resulting in receptor stabilization and signaling. This effect corresponds to an increase in epithelial to mesenchymal (EMT), cell migration, and invasion in vitro. Furthermore, we show that HUNK stabilizes EGFR expression in primary tumors, which correlates with increased levels of phosphorylation of EGFR at T654, resulting in an increase in lung metastasis in vivo. These studies are novel because they are the first to identify a substrate, EGFR, for HUNK and is the first study to show that the phosphorylation of the T654 site in EGFR regulates metastasis.

## Results

### HUNK phosphorylates EGFR at T654

**Fig. 1:**
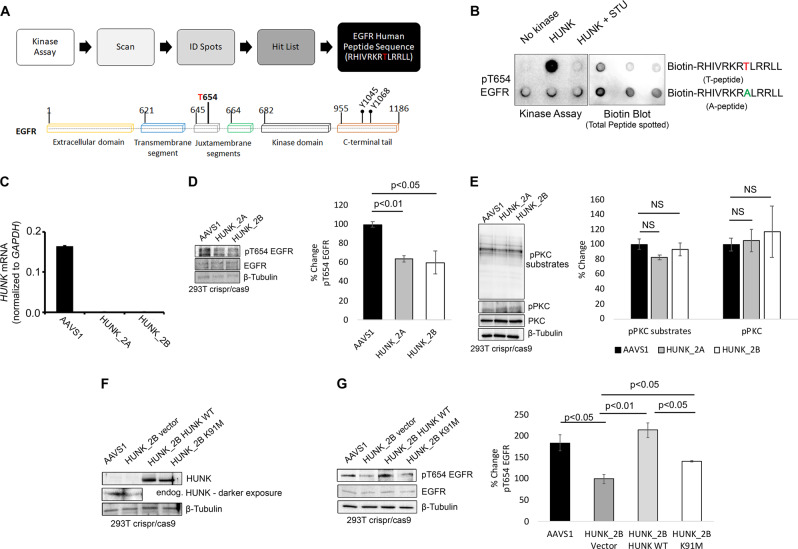
HUNK directly phosphorylates EGFR on threonine 654 (T654) residue. **a** A substrate screen was performed using a peptide microarray (JPT). 293T cells were transfected with a Flag-tagged HUNK wild type (Flag-HUNK WT), and Flag-HUNK WT was isolated using a flag peptide elution method. The eluted kinase was then applied to the peptide array to evaluate kinase activity toward spotted peptides, with an EGFR peptide as a hit. **b** Dot blot kinase assay using an EGFR peptide RHIVRKR**T**LRRLL (T-peptide) and RHIVRKR**A**LRRLL (A-peptide) as the substrate for recombinant HUNK. The peptide was incubated with ATP with no kinase, with HUNK, and with HUNK and staurosporine (STU). **c** Results from qPCR showing the crispr/cas9 depleted levels of HUNK in 293T control (AAVS1) and knock-out (HUNK_2A and HUNK_2B). Western blot showing the expression levels of (**d**) pT654 EGFR (HUNK_2A *p* < 0.01; HUNK_2B *p* < 0.05), total EGFR (**e**) pPKC, pPKC substrates, and total PKC in 293T crispr/cas9 cells. Graphs shows mean ± SEM (*n* = 3). 293T HUNK_2B cells were transfected with vector, HUNK WT, and kinase dead HUNK (HUNK K91M). Western blot showing the expression levels of (**f**) HUNK, (**g**) pT654 EGFR (AAVS1 *p* < 0.05; HUNK_2B HUNK WT *p* < 0.01; HUNK_2B K91M *p* < 0.05), and total EGFR in 293T HUNK_2B transfected cells. All graphs show mean ± SEM (*n* = 3)

We performed a HUNK substrate screen using an annotated peptide microarray spotted with 720 peptides containing known phosphorylation sites in human proteins. A total of 293T cells were transfected with a Flag-tagged HUNK wild-type (Flag-HUNK WT) followed by anti-Flag immunoprecipitation and elution with Flag peptide. The eluted kinase was applied to the peptide array with ATP [γ-^32^P] to evaluate kinase activity toward the spotted peptides. The results showed that HUNK phosphorylated a peptide containing the sequence RHIVRKRTLRRLL [24]. This peptide sequence maps to T654 and surrounding amino acids in the human EGFR protein (Fig. 1a). To confirm that HUNK directly phosphorylates this site, we performed an in vitro dot blot kinase assay using recombinant HUNK isolated from Sf9 cells, and biotin-tagged peptides containing the EGFR sequence RHIVRKRTLRRLL (T-peptide), or the same sequence but with an alanine in place of the threonine- RHIVRKRALRRLL (A-peptide) as substrates. The peptides were incubated with ATP either in the absence or in the presence of HUNK. We also used staurosporine (STU) as a pharmacological tool to inhibit HUNK kinase activity, since prior studies show STU to bind and inhibit HUNK [25, 26]. The kinase reactions were dotted onto nitrocellulose membrane and blotted with a phospho-specific antibody for T654 EGFR (pT654). HUNK phosphorylated the T-peptide, but not the A-peptide, and STU prevented T-peptide phosphorylation (Fig. 1b, left panel). We additionally blotted for biotin, using streptavidin-HRP, to confirm the presence of substrate in each reaction (Fig. 1b, right panel). These results indicate that HUNK directly phosphorylated EGFR at T654, identifying EGFR as the first HUNK substrate.

Next, we generated HUNK-deficient 293T cells using Crispr/Cas9 with two individual sgRNAs to deplete *HUNK* and a control cell line that had a sgRNA targeted to AAVS1. Loss of HUNK was quantitated using quantitative real-time PCR (Fig. 1c). We confirmed HUNK depletion decreased pT654 EGFR (Fig. 1d). Since PKC was reported to phosphorylate EGFR at T654 [24], we evaluated if PKC activation or expression was affected by altering HUNK. There was no change in the expression of PKC, phospho-PKC (pPKC), or phosphorylation of PKC substrates (pPKC substrates) (Fig. 1e). However, residual pT654 EGFR in HUNK sgRNA targeted cells could be due to PKC activity, which is present in HUNK depleted cells. To determine if we could rescue the phosphorylation of EGFR at T654 in the HUNK-deficient cells, we transfected Flag-HUNK WT and kinase-inactive Flag-HUNK K91M into the HUNK_2B 293T cells (Fig. 1f) and found the addition of Flag-HUNK WT into HUNK_2B 293T cells rescued the phosphorylation of EGFR at T654, whereas expression of Flag-HUNK K91M did not (Fig. 1g). Taken together, these data strongly suggest that HUNK kinase activity directs the phosphorylation of EGFR at T654.

### HUNK regulates EGFR stability leading to downstream signaling, increased EMT, cell migration, and invasion

**Fig. 2:**
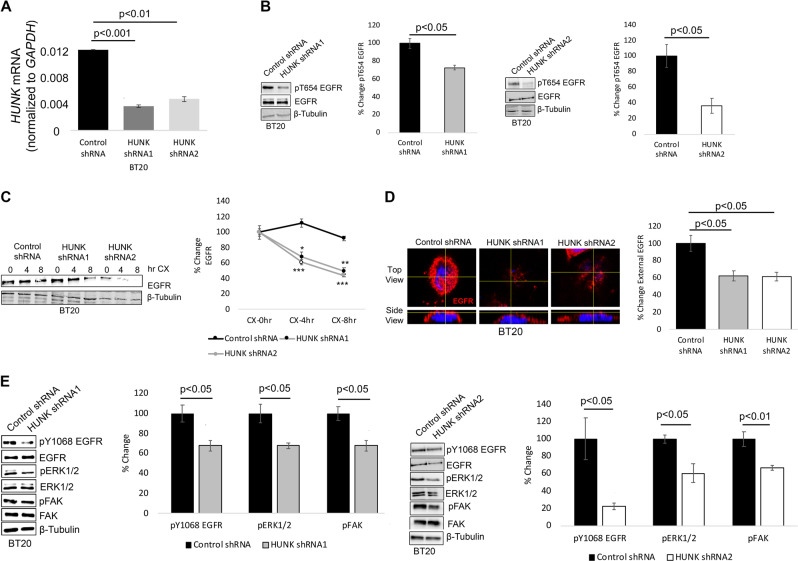
HUNK regulates pT654 EGFR and EGFR-directed metastatic signaling in BT20 cells. **a** qPCR results showing the level of *HUNK* knock-down in BT20 cells with control, HUNK shRNA1 (*p* < 0.001), and HUNK shRNA2 (*p* < 0.01). **b** Western blot showing the expression levels of pT654 EGFR and total EGFR in control, HUNK shRNA1 (*p* < 0.05), and HUNK shRNA2 (*p* < 0.05) BT20 cells. **c** Control, HUNK shRNA1, and HUNK shRNA2 BT20 cells were treated with cycloheximide (CX) over the course of 8 h, and western blot showing the expression levels of EGFR (CX-4 h HUNK shRNA1 *p* < 0.05; CX-4 h HUNK shRNA2 *p* < 0.001; CX-8 h HUNK shRNA1 *p* < 0.01; CX-8 h HUNK shRNA2 *p* < 0.001). **d** Immunofluorescence showing cell surface EGFR expression in control, HUNK shRNA1 (*p* < 0.05), and HUNK shRNA2 (*p* < 0.05) BT20 cells. Western blot showing the expression levels of (**e**) pY1068 EGFR (HUNK shRNA1 *p* < 0.05; HUNK shRNA2 *p* < 0.05), total EGFR, pERK1/2 (HUNK shRNA1 *p* < 0.05; HUNK shRNA2 *p* < 0.05), total ERK1/2, pFAK (HUNK shRNA1 *p* < 0.05; HUNK shRNA2 *p* < 0.01), and total FAK. All graphs show mean ± SEM (*n* = 3)

To investigate a role for HUNK regulation of EGFR in breast cancer, we used shRNA to target HUNK in human breast cancer cell lines that have high EGFR expression, without altering PKC activity (Supplementary Figs. 1 and 2). We engineered BT20 and MDA-MB-468 (human EGFR+ breast cancer cell lines) cells with control shRNA (targeted to firefly luciferase) or shRNA targeted to *HUNK* (HUNK shRNA1 and shRNA2) (Fig. 2a and Supplementary Fig. 2a). Downregulation of HUNK in BT20 and MDA-MB-468 cells reduced the levels of pT654 EGFR (Fig. 2b and Supplementary Fig. 2b).

Bao et al. found that the phosphorylation of EGFR at T654 impedes EGFR ubiquitination and degradation, resulting in sustained EGFR expression in the absence of the receptor ligand, epidermal growth factor (EGF) [24]. We performed a cycloheximide (CX) chase analysis to observe the role of HUNK in EGFR degradation. CX inhibits translation by impairing ribosomal translocation, and over time an unstable protein will decrease in expression whereas a stable protein will not [27]. Consistent with Bao et al. observations, HUNK down-regulation rescued EGFR degradation after CX treatment (Fig. 2c). In addition, immunofluorescence of EGFR showed that the down-regulation of HUNK reduced the amount of EGFR on the cell surface (Fig. 2d).

EGFR dimerizes with itself and other EGFR family members (e.g., HER2 and/or HER3), which causes an auto-transphosphorylation event that activates the intracellular kinase domains of the receptors, leading to phosphorylation of tyrosine (Y) residues in the C-terminal tail of each receptor [17]. Therefore, we evaluated EGFR, HER2, and HER3 for levels of trans-autophosphorylation and saw reduced phosphorylation of EGFR at Y1068 (Fig. 2e), HER2 at Y1248 (Supplementary Fig. 1b), and HER3 at Y1289 (Supplementary Fig. 1c) in BT20 cells. We saw the same decrease in trans-autophosphorylation of EGFR at Y1068 in MDA-MB-468 cells (Supplementary Fig. 2d). Levels of pY1068 EGFR were also reduced in 293 T HUNK_2B engineered cells when compared with the control cells (Supplementary Fig. 3a). Moreover, the addition of Flag-HUNK WT into HUNK_2B 293T cells rescued phosphorylation of EGFR at Y1068, whereas the addition of Flag-HUNK K91M did not (Supplementary Fig. 3a), further supporting that HUNK enhances EGFR activity. A common downstream signaling pathway of activated EGFR is the Ras-MAPK (ERK1/2) pathway [17, 28], which regulates EMT, tumor invasion, and metastasis [7]. To determine if HUNK regulates the activation of ERK1/2, we evaluated control shRNA and HUNK shRNA expressing BT20 and MDA-MB-468 cells for levels of ERK1/2 phosphorylation (pERK1/2). Concomitant with the down-regulation of pY1068 on EGFR, the down-regulation of HUNK reduced pERK1/2 (Fig. 2e and Supplementary Fig. 2d). We also evaluated the expression of pERK1/2 in 293 T control and HUNK_2B engineered cells transfected with Flag-HUNK WT and Flag-HUNK K91M and found that the addition of Flag-HUNK WT into HUNK_2B 293T cells increased the expression of pERK1/2, whereas the addition of Flag-HUNK K91M did not (Supplementary Fig. 3b). In addition to ERK1/2, studies have shown that focal adhesion kinase (FAK) can associate with activated EGFR signaling complexes to aid in cell migration [29, 30]. Therefore, we evaluated control shRNA and HUNK shRNA expressing BT20 and MDA-MB-468 cells for FAK activation and saw that the downregulation of HUNK reduced pFAK (Fig. 2e and Supplementary Fig. 2d).

**Fig. 3:**
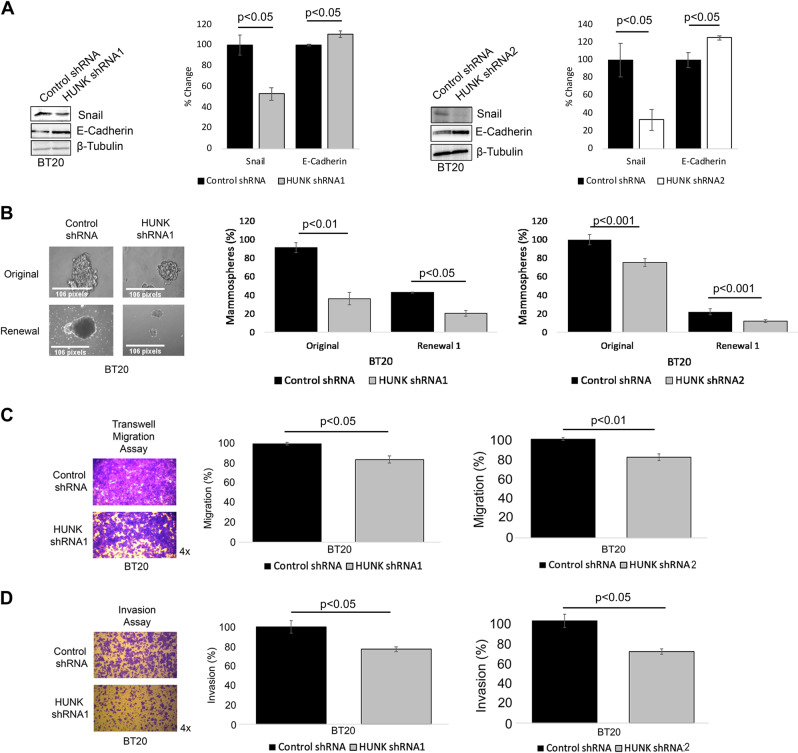
HUNK regulates EGFR-directed metastatic phenotypes in BT20 cells. **a** Western blot showing the expression levels of Snail (HUNK shRNA1 *p* < 0.05; HUNK shRNA2 *p* < 0.05), and E-Cadherin (HUNK shRNA1 *p* < 0.05; HUNK shRNA2 *p* < 0.05) in control, HUNK shRNA1, and HUNK shRNA2 BT20 cells. **b** Mammosphere formation assay of 500 control, HUNK shRNA1, and HUNK shRNA2 BT20 cells at original/day 7 (HUNK shRNA1 *p* < 0.01; HUNK shRNA2 *p* < 0.001) and renewal/day 14 (HUNK shRNA1 *p* < 0.05; HUNK shRNA2 *p* < 0.001). **c** Transwell migration (HUNK shRNA1 *p* < 0.05; HUNK shRNA2 *p* < 0.01) and (**d**) invasion (HUNK shRNA1 *p* < 0.05; HUNK shRNA2 *p* < 0.05) assays showing cell migration and invasion of 250,000 control, HUNK shRNA1, and HUNK shRNA2 BT20 cells after 24 h. All graphs show mean ± SEM (*n* = 3 for **a**–**c**, *n* = 4 for **d**)

Recently, activated EGFR signaling has been shown to promote EMT, which generates cells with stem-like properties [31] and drives cells to dissociate from the primary tumor and migrate/invade into surrounding tissues [7, 32]. Studies have shown that activation of EGFR and subsequent activation of ERK1/2 results in upregulation of Snail [33]. Furthermore, studies have shown that Snail and E-cadherin expression are inversely correlated in breast cancer [34, 35]. Therefore, we looked at expression of Snail and E-Cadherin in BT20 and MDA-MB-468 cells. The down-regulation of HUNK decreased Snail expression (Fig. 3a and Supplementary Fig. 2e), whereas E-Cadherin expression was increased (Fig. 3a and Supplementary Fig. 2e). We next performed mammosphere formation assays with our control shRNA and HUNK shRNA expressing BT20 cells and found the downregulation of HUNK decreased primary mammosphere formation as well as mammosphere renewal capacity (Fig. 3b). MDA-MB-468 cells were not tested for mammosphere formation as these cells were previously shown to be deficient for this property [36], and we were also unable to get spheres to form.

We next performed transwell migration assays to measure cell migration between control versus HUNK shRNA cells. The down-regulation of HUNK in BT20 and MDA-MB-468 cells reduced transwell cell migration (Fig. 3c and Supplementary Fig. 2f). In addition to cell migration, we evaluated cell invasion using transwell chambers with matrigel-coated membranes and found that the downregulation of HUNK decreased cell invasion in comparison to control cells (Fig. 3d and Supplementary Fig. 2g). In order to evaluate the effects of HUNK kinase activity and EGFR T654 phosphorylation on cell migration, we also performed transwell migration assays with the 293 T AAVS1 and HUNK_2B engineered cells. We found that reduction of HUNK expression reduced cell migration, and ectopic expression of Flag-HUNK WT into HUNK_2B 293T cells increased cell migration, whereas the addition of Flag-HUNK K91M did not (Supplementary Fig. 3c).

**Fig. 4:**
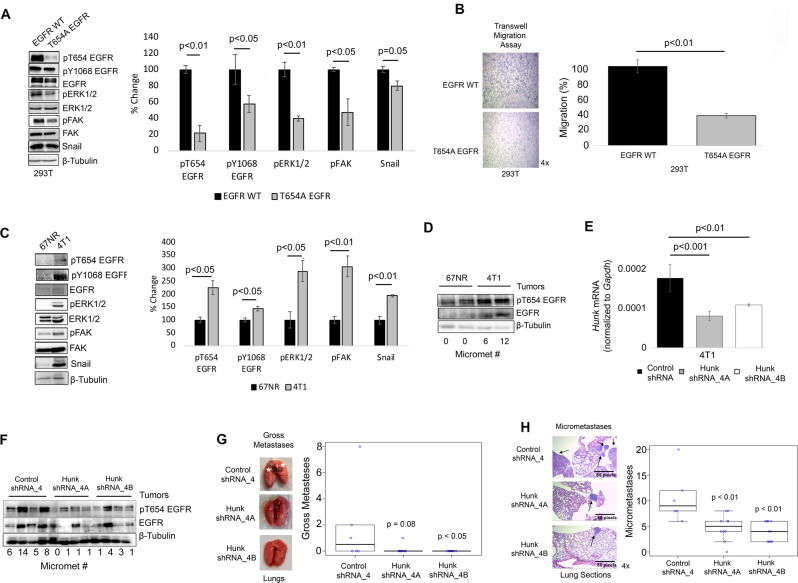
The phosphorylation of T654 on EGFR promotes breast cancer lung metastases. **a** Western blot showing the expression levels of pT654 EGFR (*p* < 0.01), pY1068 EGFR (*p* < 0.05), total EGFR, pERK1/2 (*p* < 0.01), total ERK1/2, pFAK (*p* < 0.05), total FAK, Snail (*p* = 0.05) in 293T cells transfected with EGFR WT or T654A EGFR. **b** Transwell migration assay (*p* < 0.01) showing cell migration of 150,000 293T cells transfected with EGFR WT or T654A EGFR after 24 h. **c** Western blot showing the expression levels of pT654 EGFR (*p* < 0.05), pY1068 EGFR (*p* < 0.05), total EGFR, pERK1/2 (*p* < 0.05), total ERK1/2, pFAK (*p* < 0.01), total FAK, and Snail (*p* < 0.01) in 67NR and 4T1 cells. **d** Western blot showing expression levels of pT654 EGFR and total EGFR from 67NR and 4T1 tumors. **e** qPCR results showing the level of *Hunk* knock-down in 4T1 cells with control shRNA_4, Hunk shRNA_4A (*p* < 0.001), and Hunk shRNA_4B (*p* < 0.01). **f** Western blot showing expression levels of pT654 EGFR and total EGFR from 4T1 control, Hunk shRNA_4A, and Hunk shRNA-4B tumors. Images and graphs showing (**g**) gross metastasis (identified white asterisks) in the lungs of mice bearing control, Hunk shRNA_4A (*p* = 0.08), and Hunk shRNA_4B 4T1 (*p* < 0.05) tumors and (**h**) micrometastases (identified black arrows) in the lungs of mice bearing control, Hunk shRNA_4A (*p* < 0.01), and Hunk shRNA_4B 4T1 (*p* < 0.01) tumors. Box plots show mean and spread of data set (*n* = 6–12). All other graphs show mean ± SEM (*n* = 3 for **a**, **c**–**e**; *n* = 4 for **b**)

To further investigate the roles of T654 in downstream EGFR signaling, we obtained a phospho-deficient mutant of EGFR at the T654 site (T654A EGFR) [5, 24]. We transfected EGFR wild-type (EGFR WT), and T654A EGFR into 293T cells and western blot analysis confirmed there was little to no phosphorylation of T654 on EGFR in cells transfected with T654A EGFR versus EGFR WT (Fig. 4a). Cells transfected with T654A EGFR displayed a reduction in pY1068 EGFR, pERK1/2, pFAK, and Snail (Fig. 4a) compared with cells transfected with EGFR WT. These results further confirm that the phosphorylation of T654 promotes EGFR activation and downstream signaling. We also performed transwell migration assays on 293T cells transfected with either EGFR WT or T654A EGFR, and cells transfected with T654A EGFR displayed a significant reduction in cell migration compared with cells transfected with EGFR WT (Fig. 4b). Altogether, our findings are the first to demonstrate that HUNK-directed phosphorylation of EGFR at T654 contributes to multiple metastatic phenotypes.

### Phosphorylation of T654 EGFR by HUNK promotes mammary tumor lung metastasis

To determine if HUNK regulates breast cancer metastasis in vivo, we used the 4T1 cell line, which was originally isolated as subpopulation 410.4 derived from a spontaneously arising mammary tumor in BALB/cfC3H mice, and is defined as an animal model for stage IV human breast cancer with spontaneous metastatic capability [37–40]. As our non-metastatic control, we chose to use the tumor subpopulation line 67, which was isolated from a single spontaneously arising mammary tumor from a BALB/cfC3H mouse [37], and was transfected with bacterial pSV2 plasmid to obtain geneticin-resistant 67NR cell line that is tumorigenic but will not spontaneously metastasize [41].

To first determine the significance of phosphorylation of EGFR at T654 on metastasis, we compared levels of pT654 EGFR between the two cell lines and saw a significant increase in pT654 EGFR levels in the 4T1 metastatic compared with the 67NR nonmetastatic breast cancer cells (Fig. 4c). In addition, there was a significant increase in pY1068 EGFR, pERK1/2, pFAK, and Snail in the 4T1 cells compared with the 67NR cells (Fig. 4c). We also compared pT654 EGFR levels in tumors derived from the 67NR and 4T1 tumor cells. There was less pT654 EGFR in the 67NR tumor lysates, whereas the 4T1 tumors showed higher expression of pT654 EGFR (Fig. 4d). These results suggest that the phosphorylation of EGFR at T654 correlates with and metastatic potential in vitro and in vivo.

Next, we engineered the 4T1 metastatic mammary tumor cell line to have normal (control shRNA_4) and reduced (*Hunk* shRNA_4A and *Hunk* shRNA_4B) *Hunk* expression (Fig. 4e). HUNK down-regulation reduced pY1068 EGFR, pERK1/2, and Snail (Supplementary Fig. 4a), similar to our findings in the human BT20 and MDA-MB-468 cells. In addition, downregulation of HUNK reduced mammosphere formation of 4T1 cells (Supplementary Fig. 4b), as well as cell migration and invasion (Supplementary Fig. 4c, d). These results confirmed that HUNK promotes metastatic phenotypes in the 4T1 cell line.

Next, control and *Hunk* shRNA 4T1 cells were orthotopically introduced into the abdominal mammary gland of Balb/c mice and monitored for in vivo tumor growth and lung metastasis. There was no significant difference in tumor growth between experimental groups (Supplementary Fig. 5a), consistent with previous findings [18]. Western blot analyses showed that tumors derived from *Hunk* knockdown 4T1 cells had reduced overall expression of pT654 EGFR as well as total EGFR expression compared with control cells (Fig. 4f). We observed a decrease in gross visible metastases in lungs from animals injected with 4T1 *Hunk* shRNA expressing cells compared with those derived from control cells (Fig. 4g). In addition, lungs derived from mice injected with 4T1 *Hunk* shRNA expressing cells had reduced numbers of lung micrometastases when analyzed by H&E staining (Fig. 4h). These results suggest that HUNK promotes breast cancer lung metastasis.

### Pharmacological inhibition of HUNK reduces mammary tumor lung metastasis

**Fig. 5:**
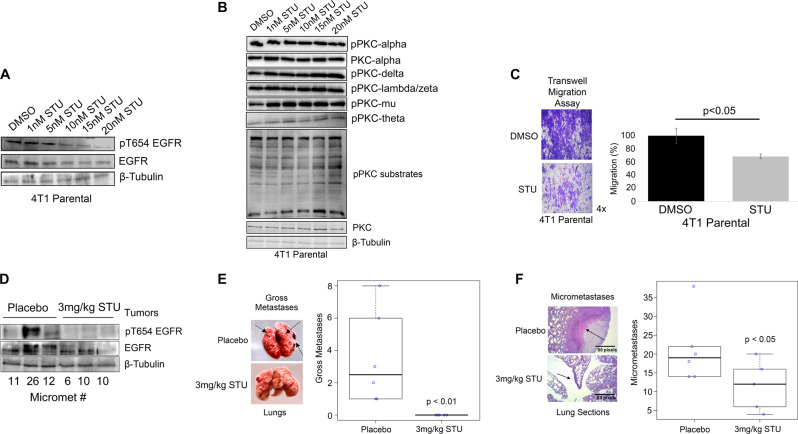
The pharmacological inhibition of HUNK impairs breast cancer lung metastasis. Western blot analyses showing the STU dose-response effects on expression of (**a**) pT654 EGFR, total EGFR, (**b**) pPKC-alpha, PKC-alpha, pPKC-delta, pPKC-lamda/zeta, pPKC-mu, pPKC-theta, pPKC substrates, and total PKC in STU dose-response in 4T1 parental cells. **c** Transwell migration assay (*p* < 0.05) showing cell migration of 50,000 cells after 24 h of 4T1 parental cells pre-treated with either DMSO or 25 nM STU. Graphs shows mean ± SEM (*n* = 4). **d** Western blot showing the expression levels of pT654 EGFR and total EGFR in 4T1 parental tumors treated with placebo or 3 mg/kg STU. Images showing (**e**) gross metastasis (*p* < 0.01) (identified black arrows) and (**f**) micrometastases (*p* < 0.05) (identified black arrows) on the lungs of mice treated with placebo or 3 mg/kg STU. Box plots show mean and spread of data set (*n* = 6–12)

Prior studies show STU to bind and inhibit HUNK [25, 26]. Therefore, we performed a dose-response analysis of STU in 4T1 cells to test the effect on phosphorylation of EGFR at T654. Increasing STU concentrations decreased the expression of pT654 EGFR (Fig. 5a), while causing no change in the activity of PKC (Fig. 5b). When cells were pre-treated with either DMSO or STU and the remaining live cell plated for transwell migration analysis, drug treatment decreased cell migration when compared with DMSO treated cells (Fig. 5c). We also treated BT20 cells with STU and performed western blot analysis to determine the effect of STU treatment on pT654 EGFR and PKC activity. STU treatment significantly decreased pT654 EGFR levels compared with cells treated with DMSO (Supplementary Fig. 6a), and STU treatment did not change the activity of PKC in the BT20 cells (Supplementary Fig. 6b). Importantly, STU treatment significantly decreased BT20 cell migration when compared with DMSO, consistent with our findings in 4T1 cells (Supplementary Fig. 6c).

To investigate the effects of pharmacological inhibition of HUNK on breast cancer metastasis in vivo, we orthotopically implanted 4T1 cells into the abdominal mammary gland of BALB/c mice. Once the tumors reached an average size of ~65 mm^3^, mice were randomly divided into two treatment groups of either placebo or 3 mg/kg STU. Once the tumors reached a maximum size of ~4000 mm^3^, the tumors and lungs were harvested. STU treatment significantly reduced the expression of pT654 EGFR in tumors compared with placebo (Fig. 5d). There was also no significant difference in tumor volume between the groups (Supplementary Fig. 5b). However, STU significantly decreased the number of gross visible lung metastases (Fig. 5e) and lung micrometastases per animal (Fig. 5f). Taken all together, our results suggest that pharmacological inhibition of HUNK impairs the phosphorylation of EGFR at T654 in primary mammary tumors and consequently, impairs metastasis.

### pT654 increases with breast cancer stage and HUNK expression in human breast cancers correlates with overall survival and distant metastasis free survival

**Fig. 6:**
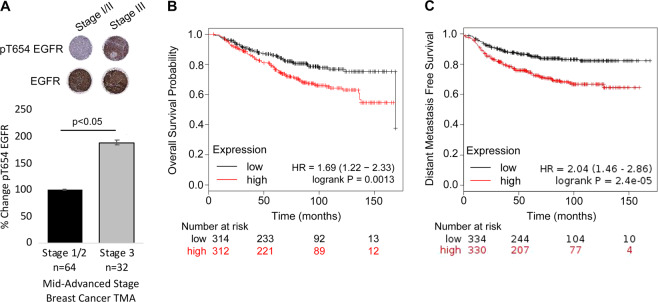
*HUNK* expression predicts overall survival and distant metastasis free survival in human breast cancers. **a** A total of 64 Stage 1/2 tissues and 32 Stage 3 tissues were analyzed for pT654 expression and graphs shows mean ± SEM. The Affimetrix gene probe for *HUNK* (1555935_s_at) was used for analysis with all clinical data available the selected (e.g., all subtypes). For OS, a total of *n* = 314 patients were scored as “low” *HUNK* and *n* = 312 were scored as “high” *HUNK*. The online tool automatically removed redundant samples, excluded any biased arrays, and did not included any proportional hazard assumptions. The probe expression range was classified by the online tool as 2–1074 with a cutoff value of 84 used for analysis. **b** For DMFS, a total of *n* = 334 patients were scored as “low” *HUNK* and *n* = 330 were scored as “high” *HUNK*. In this analysis, the probe expression range was classified by the online tool as 2–1074 with a cutoff value of 91 used for analysis

**Fig. 7:**
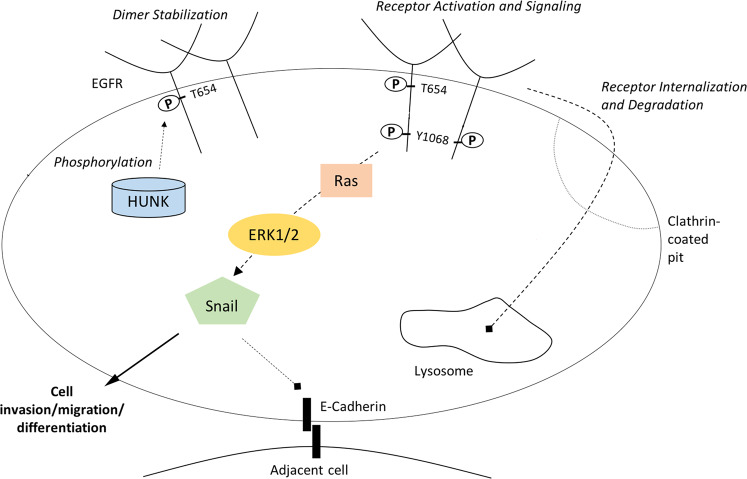
HUNK promotes breast cancer metastasis through the phosphorylation of EGFR at T654. HUNK directly phosphorylates EGFR at T654 causing impaired EGFR degradation and sustained EGFR-directed metastatic signaling in breast cancer

To further confirm our overall observations, we performed an analysis using a TMA containing samples from triple-negative breast cancer patients with stage I–III breast cancers, to analyze the levels of pT654 EGFR throughout different stages of disease. We observed a significant increase in pT654 EGFR expression in Stage 3 versus Stage 1/2 tumors (Fig. 6a and Supplementary Fig. 7). In addition, we performed analyses using the Kaplan–Meier Plotter database tool (kmplot.com) to assess whether *HUNK* expression correlated with overall (OS) or distant metastasis free survival (DMFS) [42]. The Affimetrix gene probe for *HUNK* (1555935_s_at) was used for analysis with all clinical data available selected (e.g., all subtypes). For OS, a total of *n* = 314 patients were scored as “low” *HUNK* and *n* = 312 were scored as “high” *HUNK*. The online tool automatically removed redundant samples, excluded any biased arrays, and did not include any proportional hazard assumptions. The probe expression range was 2–1074 with a cutoff value of 84 used for analysis. These parameters showed that high *HUNK* expression corresponded with decreased OS (Fig. 6b). For DMFS, a total of *n* = 334 patients were scored as “low” *HUNK* and *n* = 330 were scored as “high” *HUNK*. In this analysis, the probe expression range was 2–1074 with a cutoff value of 91 used for analysis. These parameters showed that high *HUNK* expression corresponded with decreased DMFS (Fig. 6c). Altogether, we have shown that HUNK promotes metastasis by phosphorylating EGFR, resulting in downstream signaling events that drive metastatic behavior of breast cancer cells (Fig. 7).

## Discussion

The majority of breast cancer-related deaths are not due to primary tumor growth, but rather the metastatic spread of the primary tumor to distant organs [43]. Since metastasis is the cause of most cancer-related deaths, it is important to identify the sequence of events that leads to the development of cancer cell invasion and metastasis. EGFR over-expression has been linked to metastatic phenotypes and metastatic breast cancers are considered resistant to EGFR inhibitors, which have not been clinically effective at reducing metastasis or overall survival in breast cancer patients [1, 17]. This could be due to increased metastatic potential of these tumors, but nonetheless, emphasizes the need for new therapeutic targets in patients that fail anti-EGFR therapies.

We found that through the phosphorylation of EGFR at T654, HUNK significantly increased metastatic phenotypes in breast cancer cells and mammary tumor metastasis in vivo. Our studies are consistent with other studies that demonstrate that the phosphorylation of EGFR at T654 maintains receptor stability in vitro, and EGFR overexpression enhances breast cancer metastasis in vivo [5, 24]. However, we are the first to show that phosphorylation of EGFR at T654 corresponds to increased breast cancer metastasis in vivo. In addition, we are the first to show that pT654 EGFR expression increases with higher human breast cancer stages (i.e., stage 3 » stage 1 and 2 breast cancers).

In addition, we have previously shown that STU inhibited HUNK’s kinase activity, and can therefore be used as an experimental tool to study HUNK activity [26]. We found that STU treatment of animals orthotopically transplanted with 4T1 cells reduced the phosphorylation of EGFR at T654 in primary mammary tumors, which corresponded to a decrease in EGFR expression and lung metastases. Thus, this is the first study to show that the pharmacological inhibition of HUNK reduces metastatic potential of breast cancer cells. Furthermore, we are the first to identify a kinase specific molecular mechanism for HUNK in driving metastasis and delineate the signaling pathway that HUNK acts through during this process. Our data clearly shows that pharmacological inhibition of HUNK supports this mechanism, which also has not been previously determined. While a prior publication demonstrates that pharmacological targeting of HUNK can impede tumor growth, those studies were performed in a resistant HER2-positive (HER2+) breast cancer cell model and not a metastatic breast cancer model [26]. This point is important to note because the fundamental mechanism for HUNK in HER2+ breast cancer is different from metastasis since tumor growth is not affected by HUNK targeting in metastatic models [18]. Whether this is due to differential signaling regulation or a cell type difference is yet to be determined.

Our data shows that PKC is not inhibited in 4T1 cells at the doses used to impair phosphorylation of EGFR at T654. Although prior studies demonstrate a low nM concentration range for inhibition of PKC isoforms by STU, this has not been evaluated in 4T1 cells prior to the present study. Therefore, it is possible that STU has a cell type or context specific affinity for HUNK in cells where HUNK is the predominant kinase acting on EGFR at T654. In addition, HUNK activity toward EGFR is likely supported by different cell physiological conditions that PKC activity toward EGFR, which requires calcium-dependent signaling [24]. Our findings support this notion as none of the six isoforms of PKC that we evaluated for activation after STU treatment showed a decrease in phosphorylation. Consequently, it is reasonable to speculate that STU did not inhibit PKC under the experimental conditions used to evaluate pT654 EGFR in 4T1 cells.

In summary, we show that the phosphorylation of EGFR at T654 corresponds to increased metastatic potential in vitro and in vivo, and correlates with higher stage human breast cancers. Most importantly, this is the first study to highlight EGFR T654 phosphorylation as a mechanism for breast cancer metastasis, and the first to show that pharmacological inhibition of HUNK impairs breast cancer metastasis. It is exciting to speculate that the phosphorylation of this residue could coincide with de novo EGFR inhibitor resistance in metastatic breast cancer, in particular triple-negative breast cancer that typically express high levels of EGFR. Looking forward, these findings have the potential to translate into a positive impact for the overall survival of patients with metastatic breast cancers that over-express EGFR.

## Materials and methods

### Radioactive kinase assay

For identification of HUNK substrates by peptide array, 293T cells were transfected with Flag-HUNK and the cells were lysed in buffer containing 50 mM Tris-HCl, pH 7.5, 150 mM sodium chloride, 1 mM EDTA, 1% Triton X-100 and HALT protease and phosphatase inhibitor cocktail (Thermo Scientific). Flag- HUNK was immunoprecipitated with anti-Flag M2 magnetic beads (Sigma, M8823) and eluted with 0.25 µg/ml Flag peptide (Sigma). Eluted Flag-HUNK WT was allowed to autophosphorylate in kinase buffer (20 mM HEPES, pH7.3, 2 mM MgCl_2_, 0.1 mg/ml BSA, 1 mM dithiothreitol, 100 µM ATP) for 20 min at 30 °C before addition of 50 µCi ATP-[ɣ-32P] (Perkin Elmer) and incubation for 1 hr at 30 °C with an annotated peptide array slide (JPT Peptide Technologies) containing human peptides with known phosphorylation sites, in triplicate. The peptide array was washed five times with 0.1 M phosphoric acid, dried and the radioactive image developed on a Storm 840 Phosphor Imager (Amersham). Phosphorylated peptide identification was verified by JPT Peptide Technologies.

### Dot blot kinase assay

Biotin-tagged peptides were purchased from Biomatik. HUNK underwent a 30-min preincubation step at 30 °C with 5 μM STU and ATP before peptides were added. Kinase reactions were incubated at 30 °C for 15 min, and part of the reaction was dotted on 0.2 μm nitrocellulose membrane (Biorad) using bio-dot microfiltration apparatus (Biorad), probed with a phospho-specific antibody to T654 on EGFR (pT654 EGFR), and developed on the Protein Simple FluorChem-R imaging system using HyGLo chemiluminescent detection reagent (Thomas Scientific). The biotin tag to assess total peptide was probed for with streptavidin-HRP.

### Immunofluorescence

Cells were plated and allowed to incubate on coverslips overnight. The following day the cells were fixed to coverslips using 4% Paraformaldehyde (PFA) Solution (Thermo Scientific). A permeablization step was not performed to preserve cell surface EGFR. After fixation, primary antibody EGFR (528) (Santa Cruz) antibody was used, followed by secondary antibody Alexa Fluor 594 anti-mouse IgG (Life Technologies). After antibody incubations, the coverslips were mounted to slides using ProLong Diamond Antifade Mountant with Dapi (Invitrogen). The slides were allowed to dry overnight (in the dark) at room temperature, and images were acquired with a Leica confocal microscope.

### Mammosphere formation assay

Mammosphere media was composed of a 1:1 mix of DMEM (Corning) and Hams F-12 (Corning) media supplemented with 2% B-27 supplement (Gibco), 20 ng/ml EGF (Sigma), 20 ng/mL FGF-basic (Invitrogen), and heparin (StemCell Technologies). Equal number of cells were plated in ultralow attachment plates at low density. After 7 days, mammosphere counts were recorded and images were acquired with Labomed iVu 5100 light microscope and PixelPro software. For secondary renewal analysis, mammospheres were collected and incubated with 0.05% trypsin for 5 min to produce single cell suspension. The cells were plated at the original density, and allowed to grow an additional 7 days, imaged and quantified.

### Transwell migration and invasion assays

Equal number of cells were plated in the top of the transwell inserts (VWR or Corning) or Matrigel coated invasion chamber inserts (Corning) in serum-free media. Normal growth media was placed in the bottom of the transwell apparatus. After 24 h, the transwell membranes were fixed in 4% PFA, and then stained with crystal violet. Images were acquired with Labomed iVu 5100 light microscope and PixelPro software, and the number of migrated cells was quantified using ImageJ software.

### Animal care

Animal care and experiments were approved and executed under the guidelines of the Medical University of South Carolina IACUC. All animals were housed and cared for in the AAALAC accredited Animal Research Center at the Medical University of South Carolina, and routinely monitored by lab and veterinary staff. Animals were euthanized by isoflurane overdose in accordance with the *Guide for the Care and Use of Laboratory Animals*. Protocols were in place for early and humane endpoints in the event that an experimental animal displayed signs of illness. To determine if and when the animals should be euthanized, tumor measurements and health monitoring was performed regularly by lab and veterinary staff.

### In vivo metastasis analysis

Mice were acquired from Jackson Labs. Six to eight week-old, female, BALB/c mice underwent abdominal mammary gland injection with 50,000 4T1 control shRNA_4 cells (*n* = 6), 4T1 Hunk shRNA_4A cells (*n* = 12),or 4T1 Hunk shRNA_4B cells (*n* = 12). For STU treatment, (*n* = 18) 6–8 week-old, female, BALB/c mice were injected with 100,000 4T1 parental cells. Once the tumors reached a volume of 65 mm^3^, placebo or 3 mg/kg STU oral gavage treatments were started (*n* = 6 per treatment group, 2× per week). Drugs were resuspended in 0.5% hydroxypropylmethylcellulose, 0.1% Tween-20, and 50% DMSO (placebo).

### Tissue microarray (TMA) analysis

The TMA (BR1922) was acquired from US Biomax, Inc. The microarray contained 96 cases of invasive ductal carcinoma, with 78 cases in midstage, and 18 cases in advanced stage. Duplicate cores were present for each case, and divided into two identical 96 core arrays. One of the cores was stained with pT654 EGFR (GeneTex), and the other core was stained with EGFR (528) antibody (Santa Cruz). After staining, the pT654 EGFR and EGFR cores were view on a Hamamatsu Nanzoomer, and images of each core were captured with Aperio ImageScope software. The expression levels of pT654 EGFR and EGFR were analyzed on ImageJ software.

### Statistical analysis

Group measurements for cell-based assays were compared using a two-tailed Student’s *t*-distribution. Error bars represent the standard error of the mean. Data are representative of at least three independent experiments with at least three individual replicates or more per experiment. IC_50_ was calculated using GraphPad Prism software. Micrometastases were analyzed using negative-binomial regression, and groups were compared by estimating the relative fold changes between groups using the model’s estimated coefficients. Gross metastases were summarized descriptively, and proportions of presence or absence of metastases compared between groups using Fisher’s exact test. All tests were two-sided *α* = 0.05. Survival analysis was performed using the tools provided on kmplot.com. The high and low expression groups are determined using a median split for the patients included in the particular analysis, i.e., OS and DFMS. Differences in survival distributions between the two expression groups are compared using the log-rank test, and hazard ratios are reported with their 95% confidence intervals.

## Supplementary information

Supplementary Information

SF1

SF2

SF3

SF4

SF5

SF6

SF7
