# Correction: HUNK phosphorylates EGFR to regulate breast cancer metastasis

**DOI:** 10.1038/s41388-021-01797-3

**Published:** 2021-05-06

**Authors:** Carly B. Williams, Kendall Phelps-Polirer, Ivan P. Dingle, Christina J. Williams, Matthew J. Rhett, Scott T. Eblen, Kent Armeson, Elizabeth G. Hill, Elizabeth S. Yeh

**Affiliations:** 1 Department of Cell and Molecular Pharmacology and Experimental Therapeutics, Medical University of South Carolina, Charleston, SC USA; 2 Department of Public Health Sciences, Medical University of South Carolina, Charleston, SC USA; 3 Department of Pharmacology and Toxicology, Indiana University School of Medicine, Simon Cancer Center, Indianapolis, IN USA

**Keywords:** Mechanisms of disease, Breast cancer

Correction to: *Oncogene* (2020) **39**:1112–1124

10.1038/s41388-019-1046-5 published online 9 October 2019

Unfortunately, an error occurred in preparing Fig. 4G in the original manuscript. The same representative lung image was used for HUNK shRNA_4A and HUNK shRNA_4B. The corrected complete Fig. 4 is given below. The authors apologize for this oversight and confirm that the updated figure does not change the conclusion of the experiment described in this figure or any other part of the study.

**Fig. 4:**
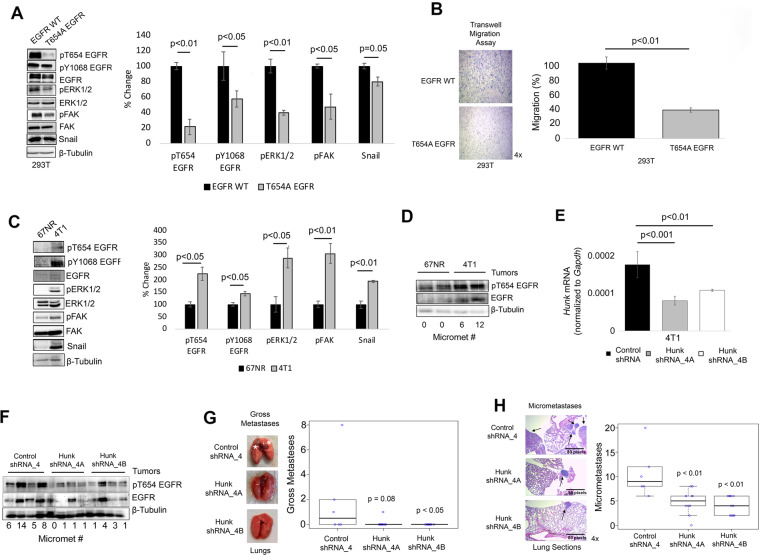
The phosphorylation of T654 on EGFR promotes breast cancer lung metastases. **A** Western blot showing the expression levels of pT654 EGFR (*p* < 0.01), pY1068 EGFR (*p* < 0.05), total EGFR, pERK1/2 (*p* < 0.01), total ERK1/2, pFAK (*p* < 0.05), total FAK, Snail (*p* = 0.05) in 293T cells transfected with EGFR WT or T654A EGFR. **B** Transwell migration assay (*p* < 0.01) showing cell migration of 150,000 293T cells transfected with EGFR WT or T654A EGFR after 24 h. **C** Western blot showing the expression levels of pT654 EGFR (*p* < 0.05), pY1068 EGFR (*p* < 0.05), total EGFR, pERK1/2 (*p* < 0.05), total ERK1/2, pFAK (*p* < 0.01), total FAK, and Snail (*p* < 0.01) in 67NR and 4T1 cells. **D** Western blot showing expression levels of pT654 EGFR and total EGFR from 67NR and 4T1 tumors. **E** qPCR results showing the level of Hunk knock-down in 4T1 cells with control shRNA_4, Hunk shRNA_4A (*p* < 0.001), and Hunk shRNA_4B (*p* < 0.01). **F** Western blot showing expression levels of pT654 EGFR and total EGFR from 4T1 control, Hunk shRNA_4A, and Hunk shRNA-4B tumors. Images and graphs showing (**G**) gross metastasis (identified white asterisks) in the lungs of mice bearing control, Hunk shRNA_4A (*p* = 0.08), and Hunk shRNA_4B 4T1 (*p* < 0.05) tumors and (**H**) micrometastases (identified black arrows) in the lungs of mice bearing control, Hunk shRNA_4A (*p* < 0.01), and Hunk shRNA_4B 4T1 (*p* < 0.01) tumors. Box plots show mean and spread of data set (*n* = 6–12). All other graphs show mean ± SEM (*n* = 3 for (**A**), (**C**–**E**); *n* = 4 for (**B**)).

The original article has been corrected.

