# Supplementary material for: HUNK phosphorylates EGFR to regulate breast cancer metastasis
Source: Oncogene. 2019 Oct 9;39(5):1112–24. doi: 10.1038/s41388-019-1046-5 (PMC6989402; doi:10.1038/s41388-019-1046-5)
Supplement: Supplementary file 1 — Supplementary Information [file 41388_2019_1046_MOESM1_ESM.docx]

**Supplementary Materials and Methods**

***Materials and antibodies***

Puromycin was purchased from Gibco. Cycloheximide was purchased from Acros Organics. Staurosporine (STU) was purchased from Selleck Chemicals. Recombinant HUNK was purchased from Life Technologies Corporation. Primary antibodies used for western blotting include: HUNK (Invitrogen), pT654 EGFR (GeneTex), pY1068 EGFR (Cell Signaling), EGFR (A-10) (Santa Cruz), EGFR (528) (Santa Cruz), pHER3 (Cell Signaling), HER3 (Thermo Scientific), pHER2 (Santa Cruz), HER2 (Cell Signaling), PKC (Santa Cruz), pPKC (Santa Cruz), pPKC substrates (Cell Signaling), PKCα (Cell Signaling), pPKCδ (Cell Signaling), pPKCθ (Cell Signaling), pPKCμ (Cell Signaling), pPKCλ/ζ (Cell Signaling), Snail (Invitrogen), E-Cadherin (G-10) (Santa Cruz), ERK1/2 (Cell Signaling), pERK (E-4) (Santa Cruz), pFAK (Cell Signaling), FAK (Cell Signaling), and anti-β-tubulin (Santa Cruz). HRP-conjugated antibodies were purchased from Jackson Laboratories. Anti-Mouse IgG DyLight 800 and Anti-Rabbit IgG DyLight 800 were purchased from Rockland.

***Cell culture***

All cells were maintained in a humidified 5% CO_2_ incubator at 37°C unless otherwise specified. Medias were supplemented with 10% heat inactivated Fetal Bovine Serum (FBS) (Gibco), L-Glutamine (Corning), and Penicillin Streptomycin solution (Corning) unless otherwise specified. 293T cells were maintained in Dulbecco’s Modification of Eagle’s Medium (DMEM) (Corning) and cells modified to deplete HUNK (via crispr/cas9) were supplemented with 0.5 μg/mL puromycin. sgRNA sequences were obtained from the human Gecko library (version 2). BT20 cells were maintained in Minimum Essential Medium Eagle (MEM) (Corning). BT20 HUNK knock-down cells were generated as previously described and maintained in media supplemented with 1μg/mL puromycin. MDA-MB-468 cells were maintained in a humidified 0% CO_2_ incubator at 37°C in Leibovitz’s L-15 Medium (Gibco). 4T1 cells were maintained in RPMI 1640 (Corning) and knock-down cell lines were supplemented with 2 μg/mL puromycin.

***RNA isolation and Quantitative RealTime PCR***

RNA was isolated from cells using the GeneJet RNA isolation kit (Thermo Scientific) according to the manufacture’s protocol. Reverse transcription was performed using BioRad iScript Supermix. RealTime PCR using PrimePCR human *HUNK* (Bio-Rad, RefSeq: NC_000021.8), mouse *Hunk* (Biorad, RefSeq: NC_000082.6), and *Gapdh* or *GAPDH* was performed using the Bio-Rad myIQ. Primers for *Gapdh* are: Forward-GCACAGTCAAGGCCGAGAAT, Reverse-GCCTTCTCCATGGTGGTGAA. Primers for *GAPDH* are: Forward-TGCACCACCAACTGCTTAGC, Reverse-GGCATGGACTGTGGTCATGAG.

***Western Blot***

All cells were lysed in buffer containing 50mM Tris-HCl, pH 7.5, 150mM sodium chloride, 1mM EDTA, 1% Triton X-100 with HALT protease and phosphatase inhibitor cocktail (Thermo Scientific). Western blot analysis was performed on the Protein Simple FluorChem-R imaging system.

**Supplementary Figure Legends**

**Supplemental Figure 1**. **HUNK’s regulation of PKC, HER2 and HER3 activity in BT20 cells.** Western blot showing the expression levels of **[A]** pPKC substrates, pPKC, PKC, **[B]** pHER2, HER2, **[C]** pHER3, and HER3 in BT20 cells.

**Supplemental Figure 2**. **HUNK regulates pT654 EGFR and EGFR-directed metastatic signaling and phenotypes in MDA-MB-468 cells.** **[A]** qPCR results showing the level of HUNK knock-down in MDA-MB-468 cells with control shRNA and HUNK shRNA1 (p<0.01). Western blot showing the expression levels of **[B]** pT654 EGFR, total EGFR, **[C]** pPKC substrates, total PKC, **[D]** pY1068 EGFR, pY1045 EGFR, total EGFR, pERK1/2, total ERK1/2, pFAK, total FAK, **[E]** Snail, and E-Cadherin in control shRNA and HUNK shRNA1 MDA-MB-468 cells. **[F]** Transwell migration (p<0.01) and **[J]** invasion (p<0.01) assays showing cell migration and invasion of 500 000 control shRNA and HUNK shRNA1 MDA-MB-468 cells after 24 hours**.** Graph shows mean ± SEM (n=4).

**Supplemental Figure 3**. **HUNK kinase activity regulates EGFR signaling and cell migration in 293T cells.** Western blot showing the expression levels of **[A]** pY1068 EGFR, total EGFR, **[B]** pERK1/2, and total ERK1/2 in 293T HUNK_2B cells transfected with vector, HUNK WT, and HUNK K91M. **[C]** Transwell migration assay (p<0.05) showing cell migration of 250 000 293T HUNK_2B transfected cells after 24 hours**.** Graph shows mean ± SEM (n=4).

**Supplemental Figure 4. HUNK regulates pT654 EGFR and EGFR-directed metastatic signaling and phenotypes in 4T1 cells.**  **[A]** Western blot showing the expression levels of pY1068 EGFR, total EGFR, pERK1/2, total ERK1/2, and Snail in control shRNA_4 and Hunk shRNA_4A 4T1 cells. **[B]** Mammosphere formation assay of 500 control shRNA_4, Hunk shRNA_4A (p<0.001), and Hunk shRNA_4B (p<0.001) 4T1 cells at day 7. Graph shows mean ± SEM (n = 16). **[C]** Transwell migration (HUNK shRNA1 p<0.05; HUNK shRNA2 p <0.05) and **[D]** invasion (Hunk shRNA_4A p<0.01; Hunk shRNA_4B p<0.05) assays showing cell migration and invasion of 250 000 control, Hunk shRNA_4A, and Hunk shRNA_4B 4T1 cells after 24 hours. Graphs shows mean ± SEM (n = 4).

**Supplemental Figure 5**. **HUNK does not regulate tumor growth of 4T1 cells.** **[A]** Tumor growth curves of control shRNA_4 (n = 6), Hunk shRNA_4A (n = 12), and Hunk shRNA_4B (n = 12) tumors over the course of 7 weeks (50 000 cells injected per mouse). **[B]** Tumor growth curves of 4T1 parental tumors treated with either placebo (n=6) and 3mg/kg STU (n=6) over the course of 4 weeks (100 000 4T1 parental cells injected per mouse).

**Supplemental Figure 6. pT654 EGFR expression and cell migration are reduced by STU treatment in BT20 parental cells.** Western blot showing the expression levels of **[A]** pT654 EGFR, total EGFR, **[B]** pPKC substrates, pPKC, and total PKC in parental BT20 cells treated with either DMSO or 50nM STU. [C] Transwell migration assay (p<0.001) showing cell migration of 50,000 cells after 24 hours of BT20 parental cells pre-treated with either DMSO or 50nM STU. Graph shows mean ± SEM (n=4).

**Supplemental Figure 7. pT654 EGFR expression is elevated in late stage breast cancers.** Mid-advanced stage breast cancer microarray IHC analyses analyzing **[A]** pT654 EGFR expression (Right panel - Hamamatsu Nanzoomer image; Left panel – ImageJ threshold for quantitation), **[B]** and total EGFR expression (Right panel - Hamamatsu Nanzoomer image; Left panel – ImageJ threshold for quantitation).
