# Supplementary figures and images for: HUNK phosphorylates EGFR to regulate breast cancer metastasis
Source: Oncogene. 2019 Oct 9;39(5):1112–24. doi: 10.1038/s41388-019-1046-5 (PMC6989402; doi:10.1038/s41388-019-1046-5)

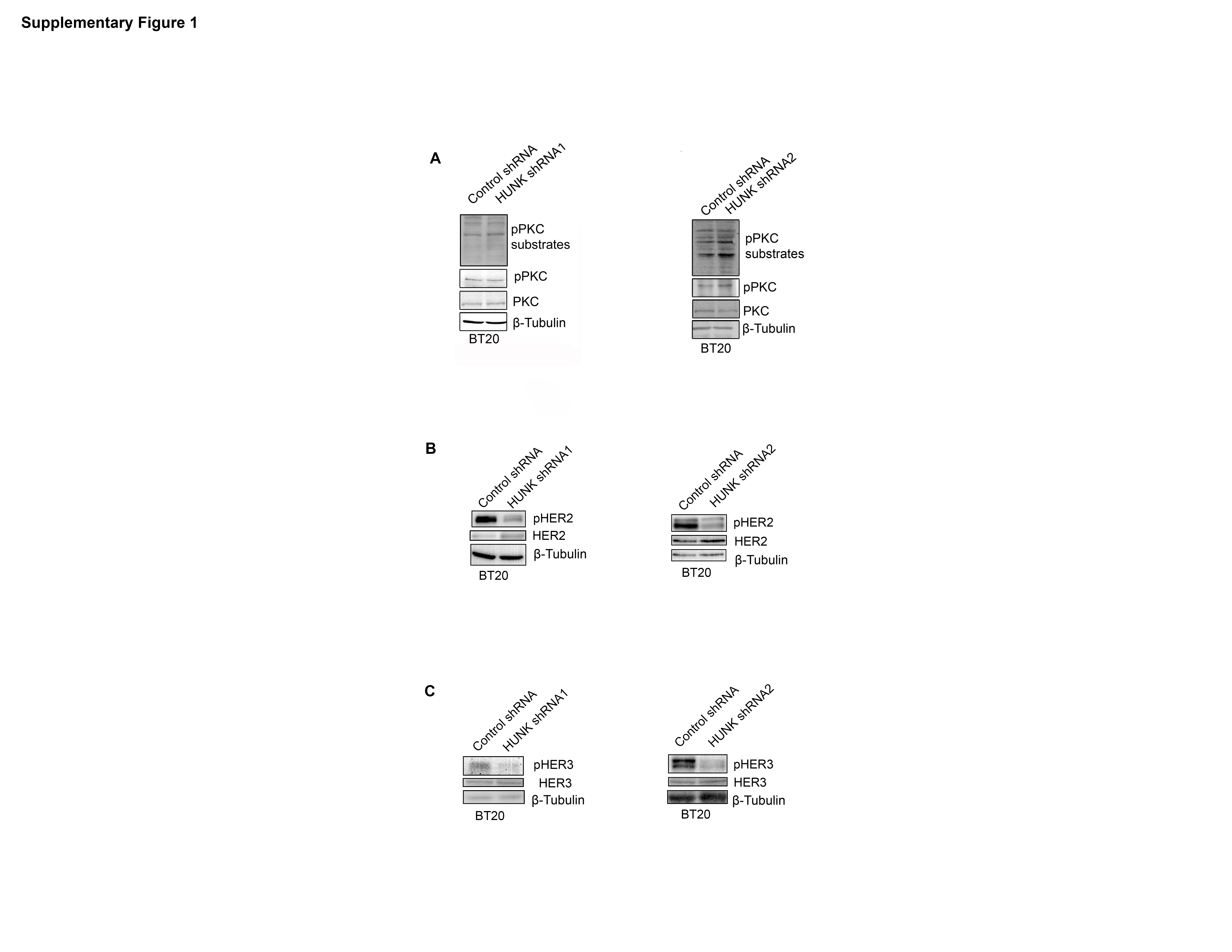

Supplement: Supplementary file 2 — SF1 [file 41388_2019_1046_MOESM2_ESM.jpg]

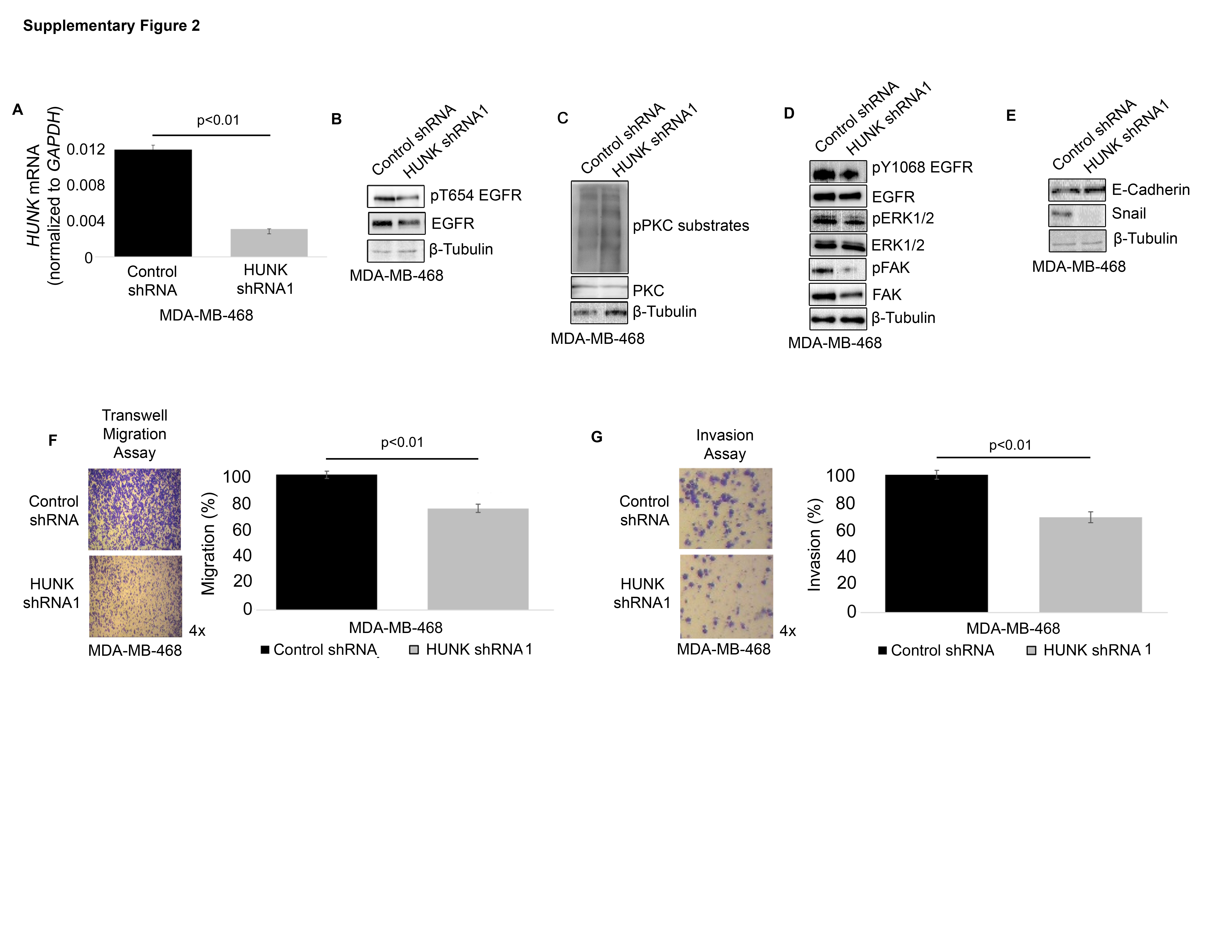

Supplement: Supplementary file 3 — SF2 [file 41388_2019_1046_MOESM3_ESM.jpg]

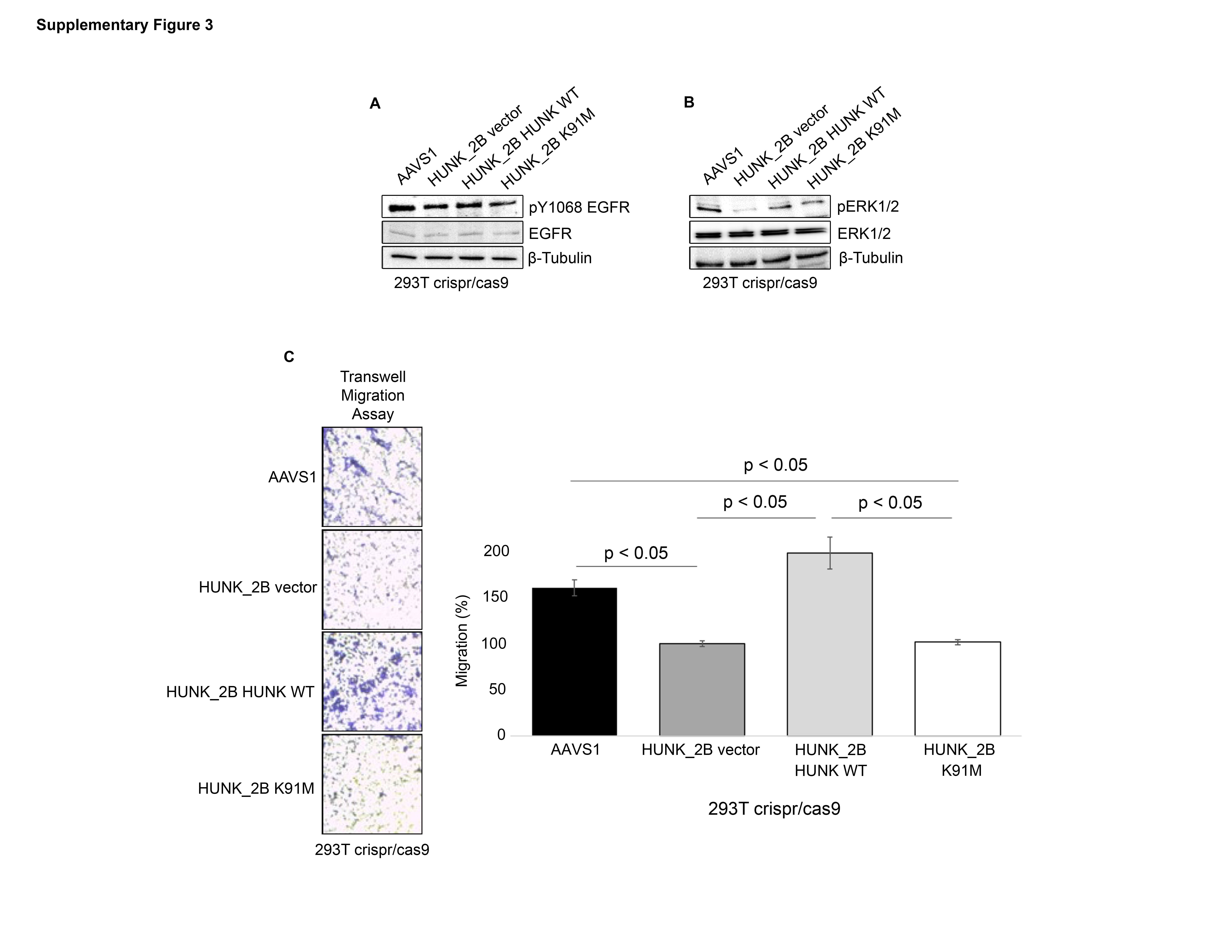

Supplement: Supplementary file 4 — SF3 [file 41388_2019_1046_MOESM4_ESM.jpg]

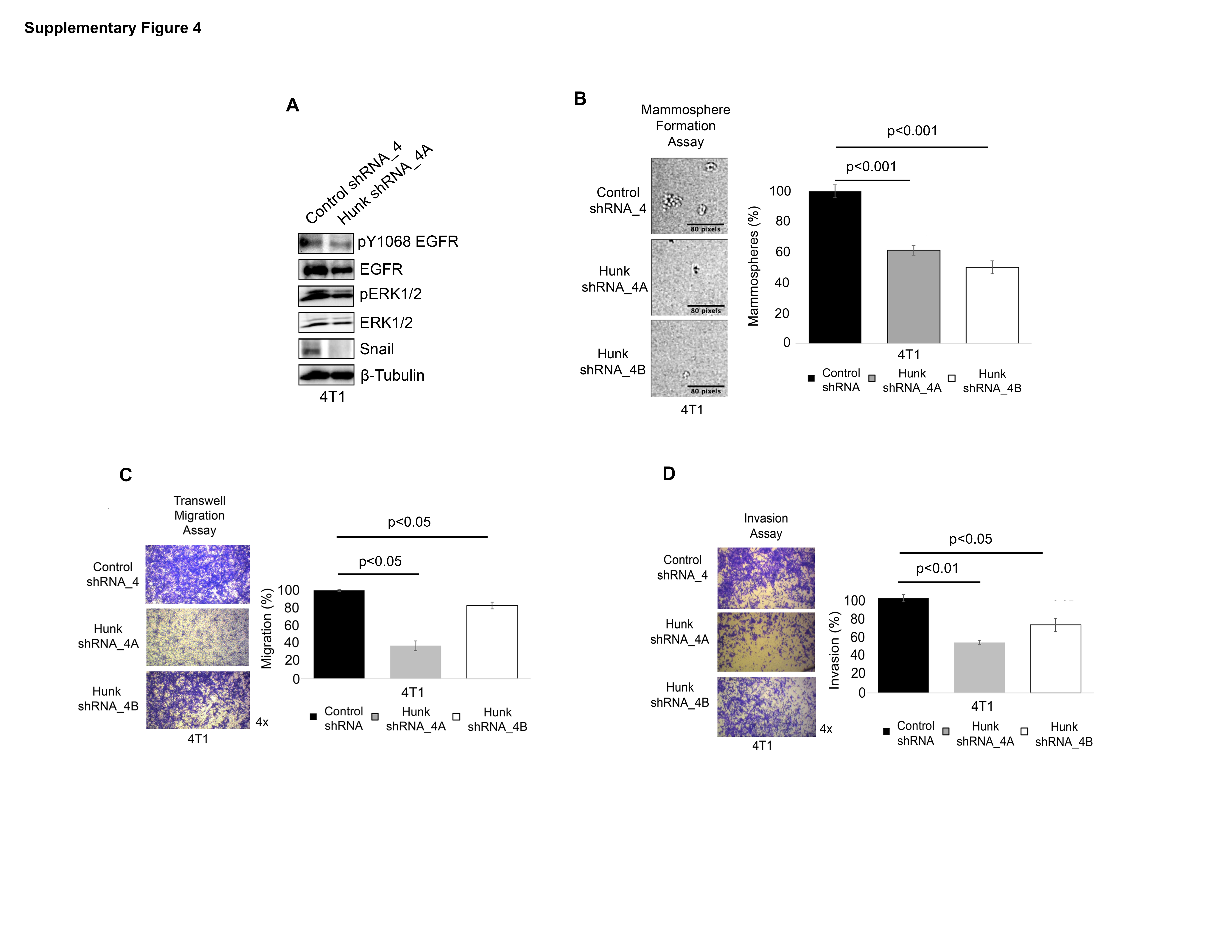

Supplement: Supplementary file 5 — SF4 [file 41388_2019_1046_MOESM5_ESM.jpg]

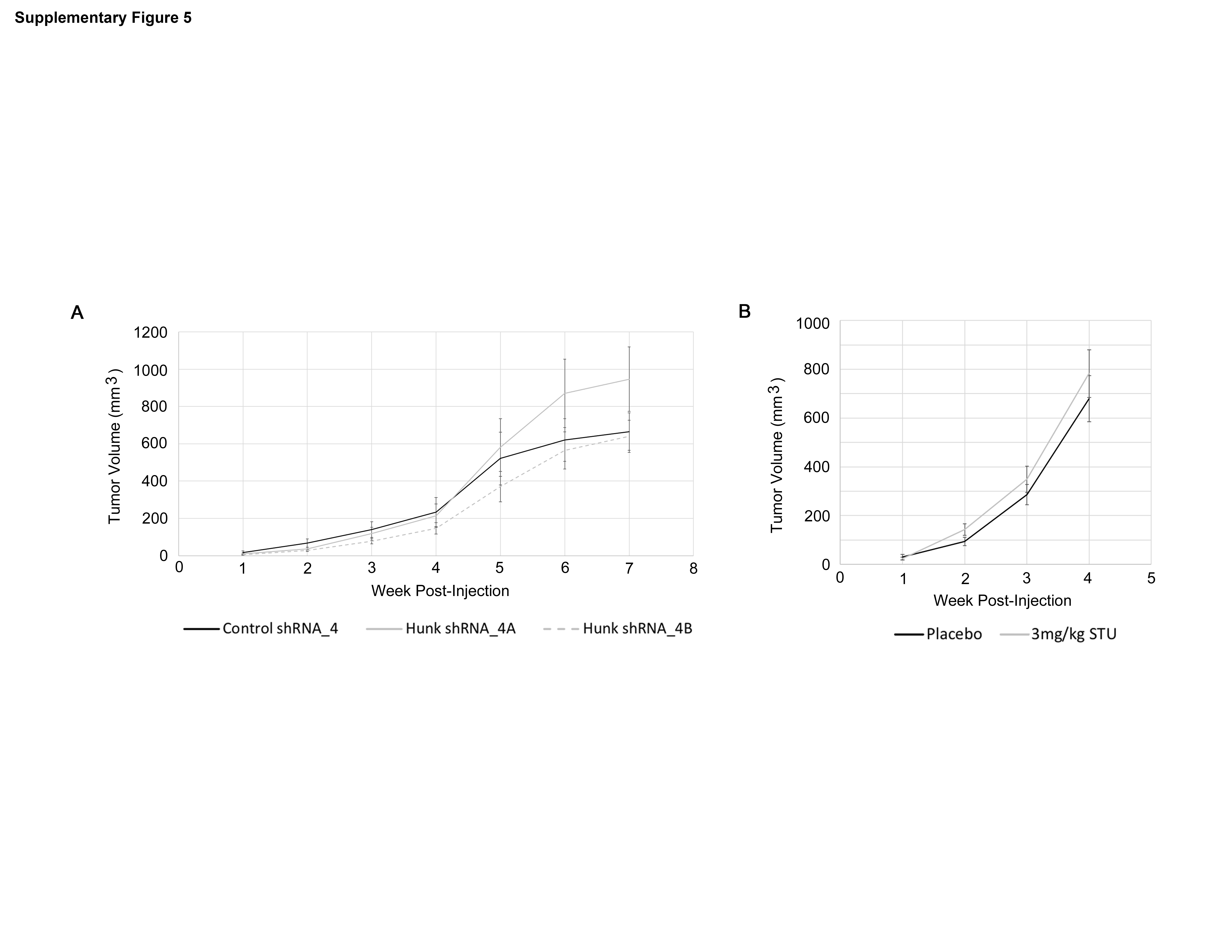

Supplement: Supplementary file 6 — SF5 [file 41388_2019_1046_MOESM6_ESM.jpg]

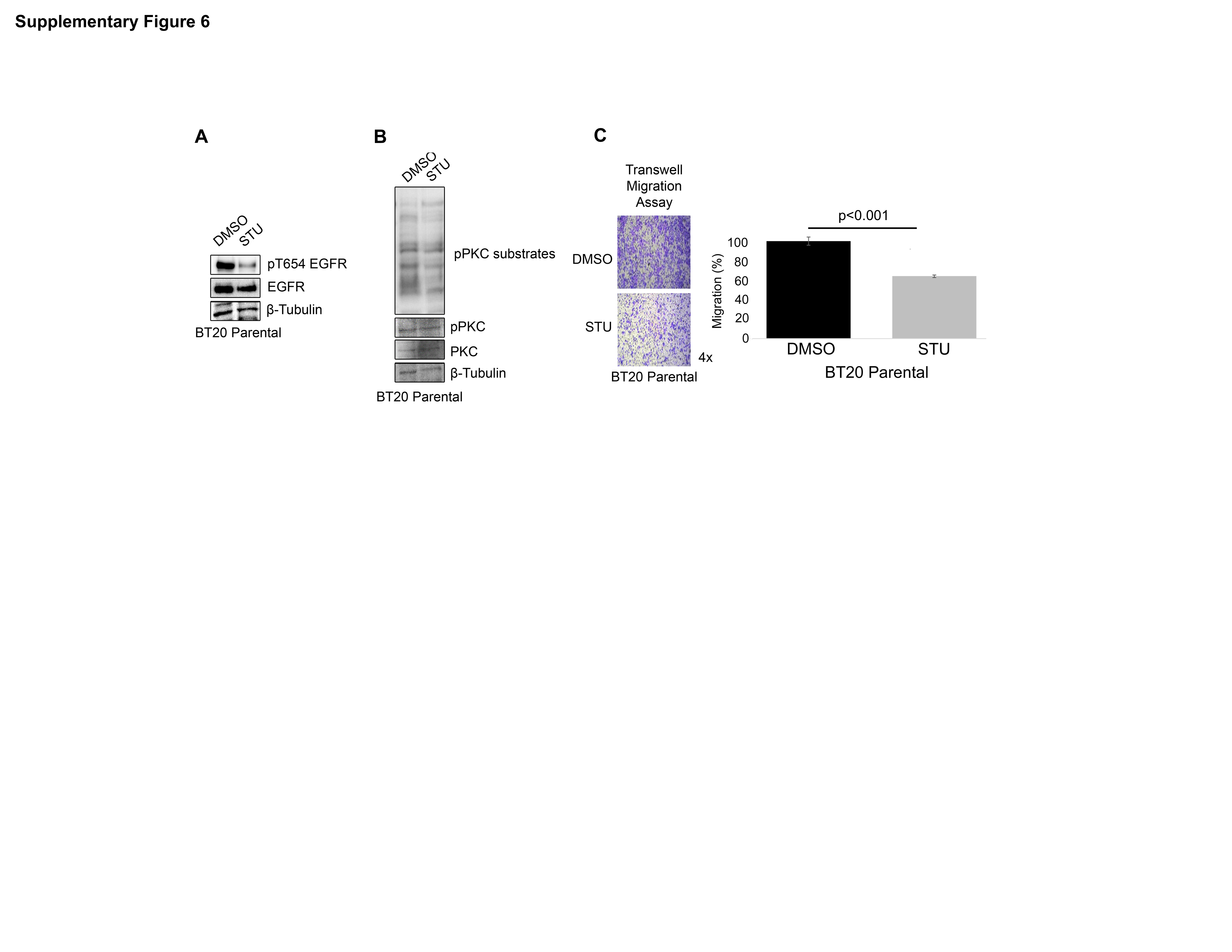

Supplement: Supplementary file 7 — SF6 [file 41388_2019_1046_MOESM7_ESM.jpg]

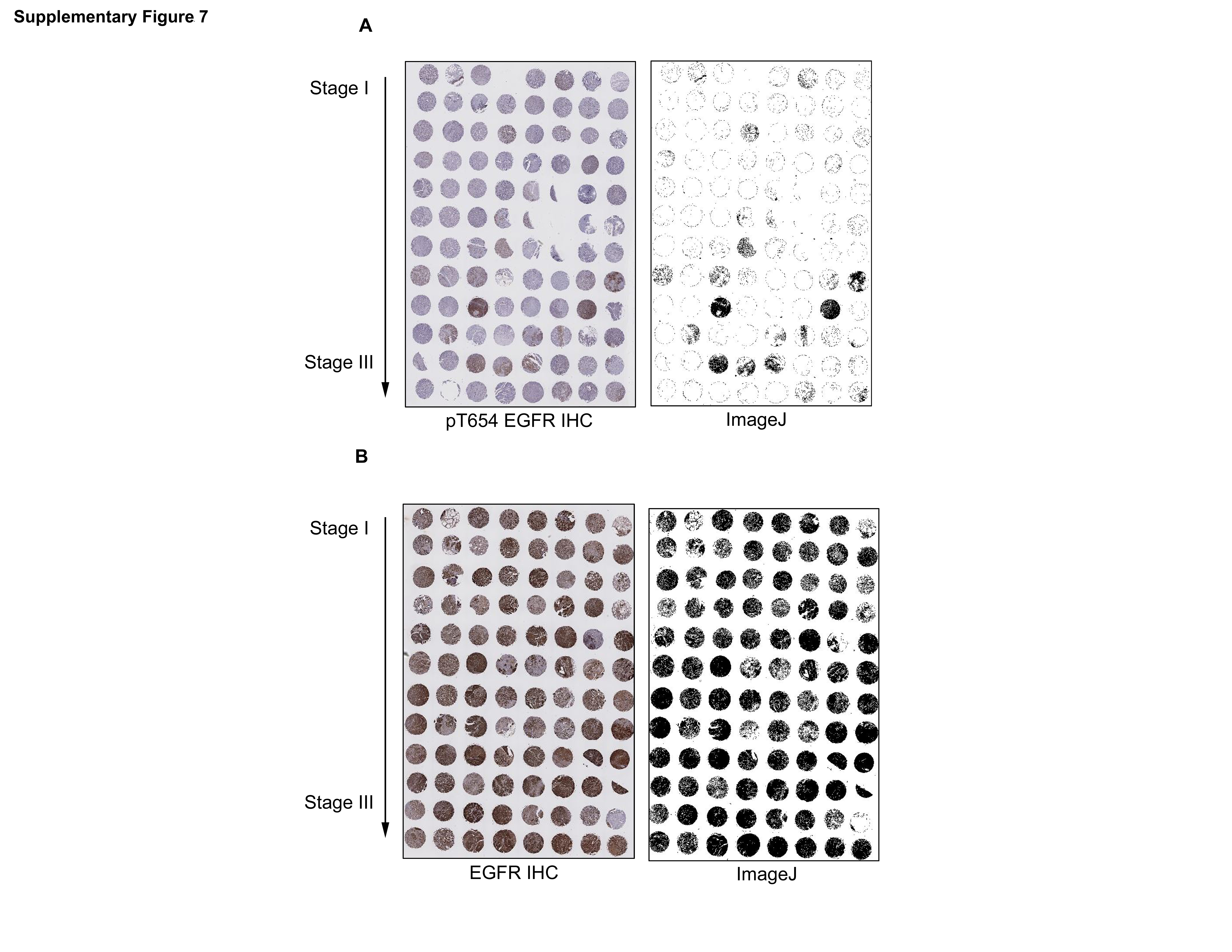

Supplement: Supplementary file 8 — SF7 [file 41388_2019_1046_MOESM8_ESM.jpg]
